# Determining the Frequency of Acquired Cystic Kidney Disease in End Stage Renal Disease Patients on Hemodialysis at Dialysis Centre of Tertiary Care Hospital

**DOI:** 10.7759/cureus.10046

**Published:** 2020-08-26

**Authors:** Muhammad Aleem, Khurram Saleem, Sana Zafar, Amina Umer, Rabia Arshad, Arsalan Nawaz

**Affiliations:** 1 Medicine, Allama Iqbal Medical College/Jinnah Hospital, Lahore, PAK; 2 Internal Medicine, University College of Medicine, The University of Lahore Teaching Hospital, Lahore, PAK; 3 Endocrinology, Diabetes and Metabolism, Services Hospital Lahore, Lahore, PAK

**Keywords:** hemodialysis, polycystic kidney disease

## Abstract

Objectives

To determine the frequency of acquired cystic kidney disease (ACKD) among patients of end-stage renal disease.

Methods

This cross-sectional study was conducted at the University of Lahore Teaching Hospital after approval from the ethical review committee. About 150 patients with end-stage renal disease fulfilling the inclusion criteria and undergoing three hemodialysis sessions per week for six months were approached. The patients underwent ultrasonography by the same consultant radiologist and the presence of acquired polycystic kidney disease was noted in the proforma. Data was stratified for age, gender and duration of hemodialysis and the chi-square test was applied.

Results

The mean age of the study participants was 47.31±9.44 years and males were majority in number with 92 (61.3%). The acquired cystic kidney disease was noted in 20 (13%) participants. There was significant difference noted in different age groups as six (6.5%) patients in the 18-40 age group and 14 (24%) patients in the 40-80 age group have acquired kidney disease (p-value=0.002). No important association between ACKD, age, and gender were found. None of these patients had evidence of renal cell carcinoma, extrarenal cysts, retroperitoneal or intrarenal hemorrhage.

Conclusion

There was a significant correlation between acquired cystic kidney disease and the duration of hemodialysis, and the chances of the development of acquired cystic kidney disease rise progressively with increasing time on hemodialysis.

## Introduction

Chronic kidney disease (CKD) is increasingly recognized as a leading public health problem worldwide [1]. It refers to conditions causing a decrease in renal function depicted by the decrease in glomerular filtration rate (GFR) ultimately leading to end-stage renal disease (ESRD) requiring dialysis. In developing countries including Pakistan, the burden of CKD and ESRD is growing and is considered to be an under-recognized public health problem in Pakistan [2]. Similarly, the risk factors and the conditions associated with chronic kidney disease leading to ESRD are also under-reported in Pakistan [3]. Acquired cystic kidney disease (ACKD) is described by the presence of numerous small to moderate size fluid-filled cysts in kidneys of ESRD patients who have no previous history of the hereditary cystic disease [4]. Diagnosis usually requires the presence of four or more cysts in both kidneys. The acquired cystic renal disorder can be differentiated from autosomal dominant polycystic renal disease (ADPKD) because the size of the kidneys is small or average relative to large kidney sizes seen in all ADPKD patients with renal failure. In addition, ACKD patients do not involve any other organs of the body and they also lack a family history of cystic disease of the kidney [5, 6].

In its early stages, acquired cystic kidney disease does not produce any symptoms but as cysts progressively increase in size and number they may be complicated by severe retroperitoneal or intrarenal hemorrhage with or without hematuria, erythrocytosis, cyst infection, and renal cell carcinoma (RCC) with distant metastasis [7]. Therefore, early detection of these complications demands regular screening in these patients.

It was initially believed that ACKD occurs in hemodialysis patients exclusively; however, later it was found that ACKD occurs in both ESRD patients undergoing treatment with peritoneal dialysis and hemodialysis [8, 9].

Studies have shown considerable variation regarding the frequency of ACKD and it ranges from 10% to 31% [10]. Moreover, only a single local study is available so far which reported its frequency of about 10%, but that study is more than a decade old while the burden of disease is rising with time [11].

The aim of this research was to determine the frequency of acquired polycystic kidney disease among patients with end-stage renal disease presenting at a tertiary care hospital. As there is limited local literature available, it becomes important to determine the frequency of ACKD as per current disease burden, diagnostic and therapeutic facilities so that clinicians get the latest information and can develop better guidelines for the screening, treatment, and prevention of acquired cystic kidney disease.

## Materials and methods

This cross-sectional study was conducted at the University of Lahore Teaching Hospital from January 1st, 2020 to June 31st, 2020. Approximately 150 patients with end-stage renal disease who met the inclusion criteria were enrolled in the study after providing written informed consent. All the patients underwent ultrasonography by the same consultant radiologist.

Inclusion criteria

Patients aged 15 to 80 years

Male and female genders

Patients of end-stage renal disease undergoing three hemodialysis sessions per week for six months at a tertiary care hospital.

Exclusion criteria

Patients not willing to participate in the study.

Patients with autosomal dominant polycystic kidney disease determined by history and medical records.

Patients with medullary sponge kidney and medullary cystic kidney determined by ultrasonography.

Sample size

A sample size of 150 cases is estimated with a confidence level of 95%, a margin of error of 5%, and the predicted percentage of acquired cystic kidney disease as 10%.

Sampling technique

Non-probability consecutive sampling was utilized.

Statistical analysis

Data was entered and analyzed using SPSS version 17.0. Numerical variable, i.e. age, was presented as mean and standard deviation. Qualitative variables such as gender, and the presence of acquired cystic renal disease were presented in the form of frequencies and percentages. Data was stratified for age, gender and duration of hemodialysis and the chi-square test was applied to check statistical significance post-stratification.

## Results

The mean age of the study participants was 47.31±9.44 years. The males were majority in number with 92 (61.3%) along with 58 (38.7%) females. Acquired cystic kidney disease was noted in 20 (13.3%) patients and absent in 130 (86.7%) patients. There was significant difference noted in different age groups as six (6.5%) patients in the 18-40 age group and 14 (24%) patients in the 40-80 age group have acquired kidney disease (p-value=0.002). (Table 1)

**Table 1:** Stratification of acquired cystic kidney disease for age

Group of age	acquired cystic kidney disease		Total	P – value
	Yes	No		
18-40 year	6 (6.5%)	87 (93.5%)	93 (100%)	0.002
40-80 year	14 (24.6%)	43 (75.4%)	57 (100%)	

 No significant difference was noted for gender (p-value=0.27). (Table 2)

**Table 2:** Stratification of acquired cystic kidney injury for gender

Gender of patients	acquired cystic kidney disease		Total	P – value
	Yes	No		
Male	14 (15.2%)	78 (84.8%)	92 (100%)	0.27
Female	6 (10.3%)	52 (89.7%)	58 (100%)	

In patients who have to be on hemodialysis for more than three years were noted to have acquired cystic kidney disease in 16 cases (16%). (Table 3)

**Table 3:** Stratification of acquired cystic kidney injury with respect to duration of hemodialysis

Group according to duration on hemodialysis	Acquired cystic kidney disease		Total
	Yes	No	
1-3 years	4 (7.8%)	47 (92.2%)	51 (100%)
More than 3 years	16 (16.2%)	83 (83.8%)	99 (100%)
Total	20 (13.3%)	130 (86.7%)	150 (100%)

## Discussion

There have been several studies to investigate the incidence, prevalence, and complications of acquired cystic kidney disease in patients with hemodialysis [12, 13]. In 1977, Dunnill and colleagues described for the first time acquired cystic disease of kidney (ACKD) after performing autopsies of 30 chronic kidney disease patients who had been treated by long-term hemodialysis. Dunnill et al. found multiple renal cysts in nearly half of these samples [14]. In our study, the prevalence of ACKD in patients on hemodialysis was 13%, which corresponds to most of the similar reports published in other parts of the world. For instance, the prevalence of ACKD in local research in Pakistan was 10%, and prevalence in Iran was 20.3% [10, 11]. Few studies have used different screening methods such as computed tomography, or autopsy, which results in a higher prevalence of ACKD as compared to when ultrasound was used [15, 16].

Increased incidence of acquired cystic kidney disease with increased duration of CKD was observed [7, 9]. In our research, acquired cystic kidney disease was found in 7.8% of ESRD patients on hemodialysis for less than three years duration and 16.2% for patients on hemodialysis for more than three years. This theory has also been observed in research by Matson et al., where the prevalence of ACKD was 10 to 20% after one to three years of hemodialysis, 40 to 60% after three to five years of hemodialysis, and more than 90% after five to ten years of hemodialysis [15]. In another study, the prevalence of ACKD was 9%, 50%, and 80% among those 54 children who had undergone peritoneal dialysis for four years or less, five to nine years, and longer than ten years, respectively [9].

Acquired cystic kidney disease occurs with nearly equal frequency in both sexes, but cystic changes are more pronounced in men. Ishikawa et al. reported that the frequency of ACKD was higher in males due to sex-related endogenous substances [13]. However, in our study, no significant difference for gender was noted in patients who developed ACKD. A study by Gnionsahe et al. observed similar insignificance of male preponderance. With respect to ethnicity, men and African Americans are at greater risk than women or Caucasians in terms of the development of ACKD [13, 17].

Renal cell carcinoma is one of the most serious complications of ACKD with an estimated incidence of 0.18% per year compared with 0.005% in the general population. Renal cancers from acquired renal cystic disease are multicentric in nearly 50% of cases and bilateral in about 10% of cases [18]. Malignancy generally develops after a long duration of dialysis (at least eight to 10 years) [19]. In our study tumors were not detected in any patient. To detect cancerous or precancerous lesions early, ultrasound of the kidney is suggested in all ESRD patients on hemodialysis for more than three years. If initial workup reveals cystic kidney disease, then computed tomography with contrast, being the more sensitive test, is recommended to evaluate for the presence of carcinoma [10, 20].

## Conclusions

There was significant correlation between acquired cystic kidney disease and duration of hemodialysis, and the chances of developing acquired cystic kidney disease rises progressively with increased time on hemodialysis. Due to certain limitations of our study, we suggest that further studies be performed on a larger sample size, spanning a longer timeframe of hemodialysis and in multiple centers to determine a cause and effect relationship.
